# High-sensitivity FEES^®^ with the professional image enhancement technology “PIET”

**DOI:** 10.1007/s00405-021-07067-y

**Published:** 2021-09-06

**Authors:** Fabian Kraus, Stephan Hackenberg, Wafaa Shehata-Dieler, Rudolf Hagen

**Affiliations:** Department of Otorhinolaryngology, Plastic, Aesthetic and Reconstructive Head and Neck Surgery, University of Wuerzburg, Josef-Schneider-Str. 11, 97080 Würzburg, Germany

**Keywords:** High-sensitivity FEES, Narrow band imaging (NBI), Professional image enhancement technique (PIET), Dysphagia, Aspiration

## Abstract

**Purpose:**

Flexible endoscopic evaluation of swallowing (FEES^®^) is a standard diagnostic tool in dysphagia. The combination of FEES^®^ and narrow band light (narrow band imaging; NBI) provides a more precise and detailed investigation method. So far, this technique could only be performed with the NBI illumination. The new version of the “professional image enhancement technique” (PIET) provides another image enhancing system. This study investigates the eligibility of PIET in the FEES^®^ procedure.

**Methods:**

Both techniques, NBI and PIET, were compared using a target system. Furthermore, the image enhancement during FEES^®^ was performed and recorded with the two systems during daily routine.

**Results:**

Performing an image enhancement during FEES^®^ is possible with both systems PIET and NBI. On the target system, the contrast of the PIET showed a brighter and a more detailed picture. In dysphagia patients, no difference between PIET and NBI was detected.

**Conclusions:**

PIET proved to be non-inferior to NBI during image enhancement FEES^®^. So far, image enhancement FEES^®^ was exclusively connected to NBI. With the PIET system, an alternative endoscopy technology is available for certain indications.

## Introduction

Narrow band imaging (NBI; Olympus Medical Systems Corp., Tokyo, Japan) is an optical technology which was first described in 1999 [1] and has been applied in different medical areas since then. An optical filter allows penetration of narrow band light with the wavelength of 415 nm (blue light) and 540 nm (green light). Depending on the soft tissue, the NBI light is absorbed in different manners. This leads to a higher contrast of vessels and surrounding mucosa. This technique is used to analyze and evaluate pathological epithelial changes [2–4]. Employing the NBI technique in a flexible endoscopic evaluation of swallowing (FEES^®^) allows a better detection of penetration and aspiration [5]. Especially, aspiration with a score of 8 on the penetration aspiration scale (PAS) according to Rosenbek [6] where parts of the bolus pass under the vocal folds into the trachea and might be overlooked. In these cases, illumination with white light is inferior to NBI due to the fact that only a few (milli)seconds with pathological findings are recorded [7, 8].

Fleischer et al. reported this positive side effect of the NBI illumination during the performance of FEES^®^ [9]. With the NBI illumination, yellow and green color turns into bright red. This emphasizes the contrast of the bolus. Due to the improved detection of smallest amounts of diffusely spread bolus, such as in laryngeal penetration and aspiration, Fleischer et al. named this procedure “high-sensitivity FEES^®^” [9]. Fleischer et al. considered that this special FEES^®^ enhancement technique can only be performed with NBI illumination. Nienstedt et al. showed a significant higher detection rate of penetration and aspiration using the NBI technique instead of white light in FEES^®^. It increases the overall reliability of FEES^®^ with a higher contrast of the bolus [5]. Even a dilution of the color up to 1:10,000 showed this effect [5]. In difficult cases, the detection of a minor but relevant penetration or aspiration is difficult and time consuming. Sometimes, only a limited number of frames in the record can be analyzed [8, 10–13].

To date, high-sensitivity FEES^®^ could only be performed with the NBI technique [5, 9]. With the new version of the “professional image enhancement technique” (PIET, Xion Medical, Berlin, Germany) another image enhancing system is available. PIET was basically developed for a better differentiation of epithelial changes. It uses a digital algorithm for an amplification of color contrast, a shift of the color spectrum and an increase of the image definition. The raw picture data is used to perform the picture analysis. To reach a better differentiation of the tissue, the red color spectrum is amplified. The quotient of the highest and the lowest brightness is enlarged to get a better luminous density (xion-medical company data information). This digital algorithm gives approximately the same change of contrast as the illumination with narrow band light.

The aim of the current study was to evaluate if PIET can be used for a high-sensitivity FEES^®^.

## Materials and methods

This retrospective study was approved by the responsible ethical committee (2,021,020,302) and was conducted according to the guidelines established in the Declaration of Helsinki (Washington 2002) for conducting clinical studies involving humans.

**Fig. 1:**
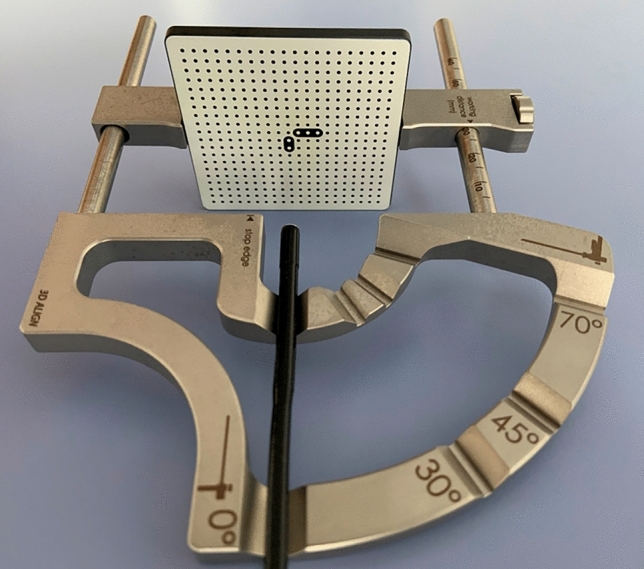
Target system with chip on the tip videoscope

For a comparison of both techniques performing high-sensitivity FEES^®^, we used a target system with a defined 3 cm distance between the tip of the endoscope and the plain of the target (Fig. 1). A high-definition flexible video-nasopharyngoscope with PIET system (video-nasopharyngoscope XN HD, Xion Medical, Berlin, Germany) and a rhino-laryngo-videoscope with NBI illumination (flexible videoendoscope ENT-VH, Olympus Medical Systems Corp., Tokyo, Japan) were sequentially attached to the target. With both endoscopes, a white balance was performed before using a standard *white balance tube*. The target was slightly moisturized with saline spray and then first sprinkled with commercially available yellow color particles (E 102). After carefully cleaning the target, green color particles (E 103) were applied. Subsequently, the target was photographed with the different chip on the tip videoscopes in white light mode and with the two different image enhancement technologies, respectively. The photographs were recorded with DiVAS (Digital Video Archive Software; Xion Medical, Berlin, Germany) and compared by an investigator with more than 10 years of NBI-experience. The difference between the red colored spots was evaluated by this experienced investigator. Furthermore, five different points of interest with different color depth were marked in both pictures by counting the pixels on the x- and y-axis. The pixels were analyzed with an image editor (GIMP—GNU Image Manipulation Program, Version 2.10.22) using the RGB color system. The RGB color system constructs all the colors from the combination of red, green and blue colors. It has integer values from 0 to 255. The higher the value the higher the color intensity. Each value can be calculated in percent. 100% stands for the highest color intensity.

In a second step, the two systems were used in the daily routine by four investigators with one, three, five and more than 10 years of experience in performing high-sensitivity FEES^®^. At the beginning of an investigation, a “plain picture” with white light and the image enhancement system was recorded. It was possible to quickly change the two illumination modes of one videoscope via a button on the handle during the postdeglutitive phase without throat clearing, coughing, or swallowing by the patient. As the pathological findings change rapidly, it was not possible to record the same findings after changing the transnasal videoscopes. Therefore, we decided to record the complete evaluation with white light and with the image enhancement system for a retrospective analysis. Furthermore, we compared the definition of NBI and PIET using the yellow colorant and green colorant in different dilutions. The different solutions were prepared using a precision balance and tested in the oral cavity in vivo and in a white cup ex vivo. The pictures were recorded and compared with the DiVAS system.

## Results

**Fig. 2:**
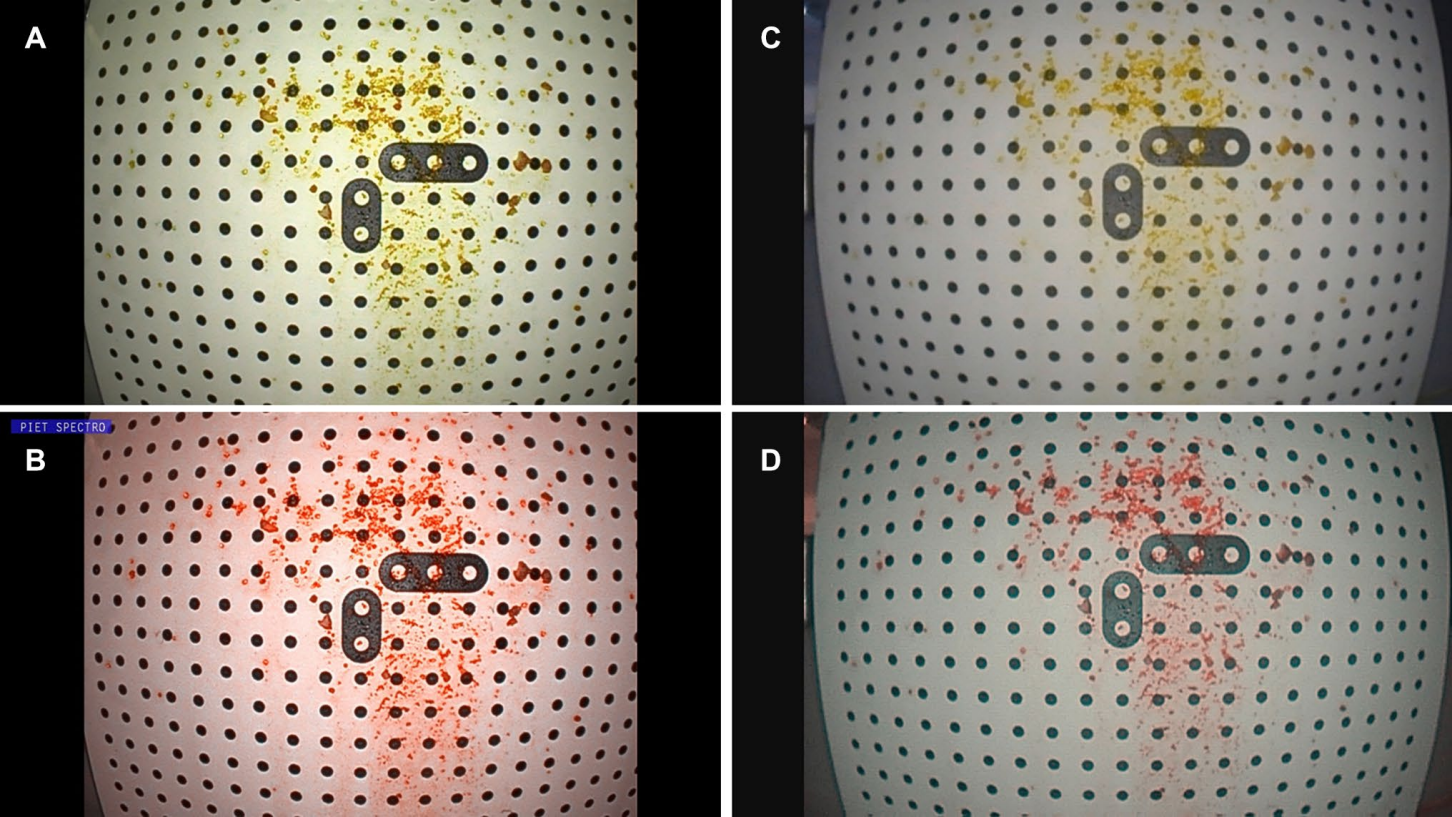
Target recorded with white light (**A**: XION, **C**: Olympus), PIET (**B**) and NBI (**D**)

An image enhancement was possible with both systems. Yellow and green colors turned to bright red. Using the target system, the contrast of the PIET system showed a brighter and a more detailed result (Fig. 2). Assessing the target with the PIET system, even slightly colored areas were detectable. With the PIET system using the measurement with the RGB color system higher values were obtained in the five defined areas (Table 1).

**Table 1:** RGB Scores for 5 different spots on the target

	Pixel 1				Pixel 2				Pixel 3				Pixel 4				Pixel 5			
	PIET		NBI		PIET		NBI		PIET		NBI		PIET		NBI		PIET		NBI	
	Val	**%**	Val	**%**	Val	**%**	Val	**%**	Val	**%**	Val	**%**	Val	**%**	Val	**%**	Val	**%**	Val	**%**
R	**132**	**51.8**	105	41,2	**208**	**81.6**	153	60.0	**196**	**76.9**	115	45.1	**204**	**80.0**	164	64.3	**110**	**43.1**	72	28.2
G	28	11	70	27.5	33	12.9	85	33.3	69	27.1	96	37.6	148	58.0	154	60.4	32	12.5	76	29.8
B	2	0.8	66	25.9	0	0.0	76	29.8	35	13.7	91	35.7	135	52.9	145	56.9	15	5.9	72	28.2

Maximum scores for “red” in bold. Range of the RGB Scale: 0–255

The dilution of 1:10,000 was detectable with both systems in the oral cavity as well as in a white cup ex vivo. A higher dilution of 1:15,000 was detectable in a white cup ex vivo but no longer in the oral cavity. A dilution higher than 1:10,000 was not clearly detectable on the mucosa. Dilution lower than 1:10,000 gave the same effects. Yellow color particles (E 102) and green color particles (E 103) offered the best results. We also tested blue color particles, but this did not show any illumination effects with PIET or NBI.

**Fig. 3:**
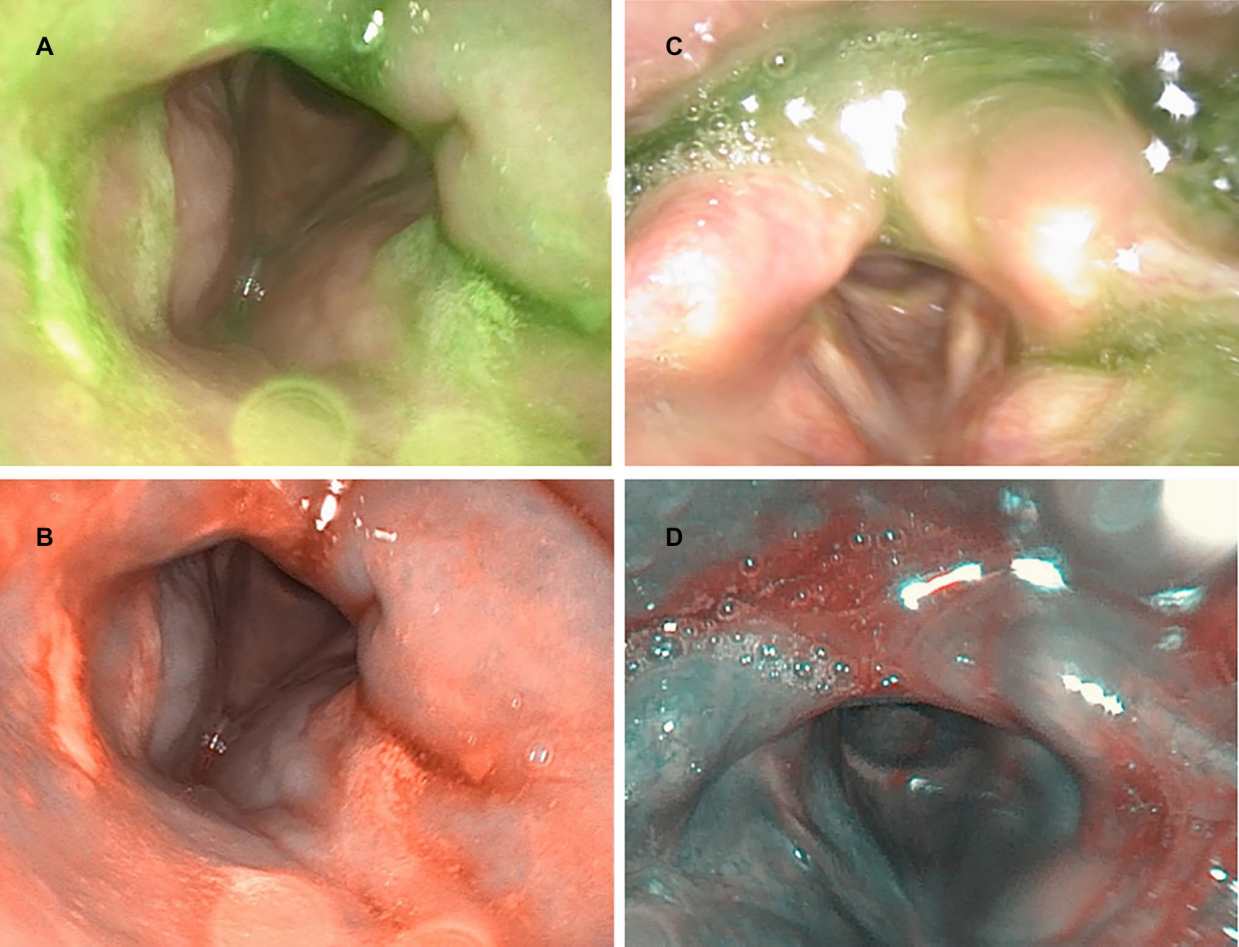
High-sensitivity FEES with white light (**A**: XION, **C**: Olympus), PIET (**B**) and NBI (**D**)

When performing a high-sensitivity FEES^®^ in dysphagia patients, both systems were able to produce a high definition of the red spots. This was even possible under minor light exposure conditions i.e., in the anterior commissure (Fig. 3) or in the trachea during a dipping maneuver. There were no differences detected between PIET and NBI.

## Discussion

FEES^®^ is a standard procedure in evaluation of dysphagia patients. Using food colorant, the visibility and the differentiation of various consistencies can be achieved. It is possible to attain a higher contrast using optical filters like the PIET or the NBI technique. To distinguish FEES^®^ with white light from FEES^®^ with optical filter amplification, this procedure was named “high-sensitivity FEES^®^” [5, 9]. This study provides evidence for the feasibility of this procedure to be done with the PIET technique and compares the findings to those done with the NBI technique.

The PIET system proved to be eligible for high-sensitivity FEES^®^. Light colored spots which could not be detected using white light, were easily identified with image enhancement systems [5, 9]. The PIET system is applicable via pressing a button on the endoscope during standard FEES^®^. It was possible to perform the same procedures as with the NBI technique.

Both image enhancement techniques are able to provide a better visualization of green and yellow-colored boli during dysphagia assessment. The comparison of the target pictures recorded with the two different systems showed, that both techniques increase the detection rate and definition of color particles. On the target, parts of faintly colored areas are better visible with PIET. Furthermore, the PIET system demonstrated higher values of the red color intensity (RGB color system) contrast compared to the NBI illumination. This means a better visualization. In vivo the best result were obtained with yellow (E 102) and green (E103) color particles with a maximum dilution of 1:10.000. Other colors and higher dilutions were not visible during image enhancement.

Using the PIET system during FEES^®^, the identification of laryngeal penetration or aspiration is at least as easy as with the NBI.

The video recordings of high-sensitivity FEES^®^ with both systems confirm the high-quality color detection even at high colorant dilution [5, 8, 9]. PIET is able to detect even micro-aspiration events such as those due to penetration of tiny bolus components in the trachea. In summary, we were able to demonstrate that in addition to the NBI, the PIET System is available to perform high-sensitivity FEES^®^ [5, 9].

With PIET as an additional available tool for high-sensitivity FEES^®^, more operators will be able to perform this diagnostic method [12, 13]. Moreover, patients with a minor compliance for an endoscopic swallowing evaluation can be reliably examined within just a few seconds. Limited frames of the record can be used for the evaluation. It is even possible to examine small children where sometimes only a few frames are available for the analysis [8]. For young residents, it is easier to evaluate FEES^®^ under difficult conditions [12, 13]. The image enhancement systems can help in different situations to get an excellent visualization of the swallowing pattern.

In some rare conditions, the combination of a stroboscopy gave further information, which is only offered by the Xion Medical system. With this system, it is possible to switch between PIET and stroboscopy modes via two buttons on the handle. The stroboscopy can provide more information about the vocal fold movement. Thus, it is possible to evaluate the complete laryngeal function.

Both image enhancement techniques are used as an online system. In some cases, the investigator realizes during review of the FEES^®^ recording that a high-sensitivity FEES^®^ would have been necessary. As PIET uses a software algorithm, it should be possible to apply “high-sensitivity FEES^®^” to the raw video data in an offline mode. This could provide more flexibility and a big advantage for offline improvement of the diagnostic ability of this tool.

The data demonstrate that PIET and NBI enhancement are suitable for the detection of pathologic findings even under difficult conditions like suboptimal illumination or in patients with a limited compliance. This facilitates the FEES^®^ procedure. Besides the one-time investment of a “chip on the tip” videoscope with *image enhancement*, no further equipment is necessary to use with this method [3, 5, 7, 9, 12, 13].

## Conclusion

Our data suggest that both techniques (PIET and NBI) are at least equivalent. PIET is able to give the same information during FEES^®^ as NBI illumination does. Both systems allow an uncomplicated switching from standard FEES^®^ to high-sensitivity FEES^®^ with comparable results.
